# Hepatitis B virus genotypes and resistance mutations in patients under long term lamivudine therapy: characterization of genotype G in Brazil

**DOI:** 10.1186/1471-2180-8-11

**Published:** 2008-01-22

**Authors:** Marcelle Bottecchia, Francisco JD Souto, Kycia MR Ó, Marcia Amendola, Carlos E Brandão, Christian Niel, Selma A Gomes

**Affiliations:** 1 Laboratório de Virologia Molecular, Instituto Oswaldo Cruz, FIOCRUZ. Av. Brasil 4365, 21045-900 Rio de Janeiro, RJ, Brazil; 2 Núcleo de Estudos de Doenças Infecciosas e Tropicais, Faculdade de Ciências Médicas, Universidade Federal de Mato Grosso, Av. Amarílio de Almeida 215, 78010-060 Cuiabá, MT, Brazil; 3 Hospital Alcides Carneiro, Fundação Municipal de Sáude de Petrópolis, Rua Vigário Correas 1345, 25720-320 Petrópolis, RJ, Brazil; 4 Hospital Universitário Gaffrée e Guinle, Rua Mariz e Barros 775, 20270-004 Rio de Janeiro, RJ, Brazil

## Abstract

**Background:**

Lamivudine is an oral nucleoside analogue widely used for the treatment of chronic hepatitis B. The main limitation of lamivudine use is the selection of resistant mutations that increases with time of utilization. Hepatitis B virus (HBV) isolates have been classified into eight genotypes (A to H) with distinct geographical distributions. HBV genotypes may also influence pathogenic properties and therapeutic features. Here, we analyzed the HBV genotype distribution and the nature and frequency of lamivudine resistant mutations among 36 patients submitted to lamivudine treatment for 12 to 84 months.

**Results:**

Half of the patients were homosexual men. Only 4/36 (11%) patients were HBV DNA negative. As expected for a Brazilian group, genotypes A (24/32 positive individuals, 75%), D (3/32, 9.3%) and F (1/32, 3%) were present. One sample was from genotype C, which is a genotype rarely found in Brazil. Three samples were from genotype G, which had not been previously detected in Brazil. Lamivudine resistance mutations were identified in 20/32 (62%) HBV DNA positive samples. Mean HBV loads of patients with and without lamivudine resistance mutations were not very different (2.7 × 10^7 ^and 6.9 × 10^7 ^copies/mL, respectively). Fifteen patients showed the L180M/M204V lamivudine resistant double mutation. The triple mutant rt173V/180M/204V, which acts as a vaccine escape mutant, was found in two individuals. The three isolates of genotype G were entirely sequenced. All three showed the double mutation L180M/M204V and displayed a large genetic divergence when compared with other full-length genotype G isolates.

**Conclusion:**

A high (55%) proportion of patients submitted to long term lamivudine therapy displayed resistant mutations, with elevated viral load. The potential of transmission of such HBV mutants should be monitored. The identification of genotypes C and G, rarely detected in South America, seems to indicate a genotype distribution different to that observed in non treated patients. Disparities in routes of transmission (genotype G seems to be linked to homosexual behavior) and in pathogenic properties (genotype C is very aggressive) among HBV genotypes may explain the presence of rare genotypes in the present work.

## Background

Hepatitis B virus (HBV) is an etiological agent of acute and chronic liver disease. Despite the introduction of HBV vaccination programs in the last decade, hepatitis B remains a largely disseminated disease, with an estimated 350 million people chronically infected around the world, and over 2 billion people who have already been infected with the virus. It is estimated that between 1 and 2 million people die annually as a consequence of the infection [1].

Based on sequence divergence in the entire genome exceeding 8%, HBV is classified into eight genotypes (A to H) (see [2-4] for reviews) that are related to their geographical origin. Most genotypes have been recently divided into subgenotypes with distinct virological and epidemiological properties. In addition, recombination among HBV genotypes increases the variability of HBV [5]. Genotype A is present in Europe, India, Africa and Americas. Genotype A is subdivided into three subgroups or subgenotypes (Aa/A1, Ae/A2 and A3). Isolates belonging to subgroup Aa/A1 have been identified in populations and descendents of African countries [6,7]. Subgenotype Ae/A2 has a European origin, whereas subgenotype A3 has been identified in Central and West Africa [8,9]. Genotypes B and C are predominant in China, Japan and Southeast Asia. Genotype D is widespread, predominating in the Mediterranean area and in the Middle East region. Genotype E appears to derive from West Africa. Genotype F is found in the aboriginal populations of the Central and South America. Genotype H has been described in Mexico and Central America [5,10]. Genotype G has been first identified in France and the United States [4], and recently detected in Mexico [11]. It seems that genotype G prevails in restricted areas in the world, possibly disseminating via particular routes of transmission [12]. Genotype G has been found in a relatively high frequency (28%) in Mexican men who have sex with men [13]. Genotype G is usually coinfected with genotype A [12] or, less frequently, with genotype H [13]. HBV genotype G is distinct from the genomes of the other seven genotypes in that it possesses a 36-nt insertion at the 5' end of the core (C) gene and translational stop codons at positions 2 and 28 in the pre-C region, preventing HBeAg synthesis [4]. Despite these stop codons, patients infected with genotype G have been found positive for hepatitis B *e *antigen (HBeAg) [14]. This could be explained by the fact that genotype G is commonly found in coinfection with genotype A. However, at least one case of monoinfection with genotype G alone has been described [15]. This could suggest that HBeAg is dispensable for viral replication. When searched for, genotype G has not been identified in several countries, and seems to be an extremely rare genotype in Japan [16].

In Brazil, genotypes A, D and F are the most prevalent [6,17,18]. In previous studies conducted in the general Brazilian- or in the Afro-Brazilian population, subgenotype A1 was the most frequent subgenotype [6,19].

Lamivudine is an oral nucleoside analogue inhibiting the reverse transcriptase activity of HIV and HBV [20]. This drug is widely used in the treatment of hepatitis B and as part of the treatment of HIV infection. A period of one year of therapy in HBeAg positive patients has been associated with HBeAg loss in 17–33% of patients and HBeAg seroconversion rates of 16–18% [21]. HBeAg seroconversion is the result of a profound suppression of viral replication, since it has only been found in patients with a reduction in HBV DNA to <10^4 ^copies/mL [21,22]. The major limitation in the use of lamivudine is the selection of resistant mutations that may arise and accumulate during therapy. These mutations usually affect the YMDD motif located in the C domain of the HBV reverse transcriptase (rt), by replacement of a methionine residue at position 204 to either valine (rtM204V, occidental population) or isoleucine (rtM204I, oriental population) [21-24]. The proportion of patients in whom YMDD variants are detectable increases with time on lamivudine therapy, rising from around 15–25% after one year of treatment to 70% by four years [21,25]. In addition, a second lamivudine resistance mutation (rtL180M), located in the B domain of rt, has been identified [26]. This second mutation is associated with prolonged lamivudine treatment. Such double mutations have notably been reported in studies performed with HIV-HBV co-infected patients [27,28]. More than 90% of the HIV-HBV co-infected patients under lamivudine treatment display the double resistant mutation rtL180M/rtM204V [28].

Hepatitis B acute exacerbation may occur after the appearance of YMDD mutants [29], accompanied by a rapid increase of viral load and serum aminotransferase levels [21]. Such exacerbations appear to be more severe than those occurring during the natural course of wild type HBV chronic infection [21]. At present, management of patients with lamivudine mutations rests on addition or replacement with other antiviral agents as adefovir dipivoxil or entecavir, which may effectively suppress the YMDD mutants [23,30,31].

The aim of the present study was to investigate the prevalence of HBV DNA and viral load variations in patients receiving lamivudine for a long period for HBV treatment, to characterize HBV genotypes, and to determine the frequency and nature of lamivudine resistance mutations. Furthermore the entire nucleotide sequences of three genotype G isolates recovered from this work were determined.

## Results

### Serological patterns and distribution of HBV genotypes

Among 36 HBsAg positive patients under long-term lamivudine treatment, only four (two male and two female) were tested negative for HBV DNA by nested PCR. All four were HIV negative, one was HBeAg positive and three were anti-HBe positive (Table 1). Thirteen patients, all men, were co-infected with HIV, 10 of them belonging to risk groups associated with sexual transmission (homosexual or bisexual).

**Table 1:** Demographic, serological and molecular data of the patients

Patient	Sex	Age	HIV	Lamivudine treatment (months)	HBe/AntiHBe	HBV load (copies/mL)	Phenotypic resistance	Lamivudine resistance mutations (rt)
*HBV DNA negative*								
16-ufmt	F	69	No	16	HBeAg	<10^2^	No	-
10-ufmt	F	60	No	14	Anti-HBe	<10^2^	No	-
35-ufmt	M	58	No	50	Anti-HBe	<10^2^	No	-
12-ufmt	M	44	No	24	Anti-HBe	<10^2^	No	-
								
*Genotype A1*								
05-hac	M	58	No	34	nd	8 × 10^4^	Yes	M204I
01-hgg	M	56	Yes	60	HBeAg	8 × 10^6^	Yes	L180M/M204V
02-hgg	M	50	Yes	84	Anti-HBe	1 × 10^6^	Yes	L180M/M204V
04-hgg	M	43	Yes	32	HBeAg	7 × 10^6^	Yes	L180M/M204V
06-hgg	M	34	Yes	72	HBeAg	1 × 10^7^	Yes	L180M/M204V
07-hgg	M	61	Yes	32	HBeAg	1 × 10^8^	Yes	L180M/M204V
10-hgg	M	46	Yes	42	HBeAg	4 × 10^5^	Yes	L180M/M204V
11-hgg	M	40	Yes	30	HBeAg	1 × 10^7^	Yes	L180M/M204I
30-ufmt	M	47	Yes	33	HBeAg	2 × 10^8^	Yes	L180M/M204V
34-ufmt	M	12	Yes	24	Anti-HBe	3 × 10^6^	Yes	L180M/M204V
03-hgg	M	35	Yes	72	HBeAg	9 × 10^5^	Yes	V173L/L180M/M204V
08-hgg	M	60	Yes	31	HBeAg	1 × 10^6^	Yes	V173L/L180M/M204V
03-hac	F	55	No	14	HBeAg	nd	Yes	No
04-hac	M	60	No	71	nd	nd	No	No
05-ufmt	M	47	No	47	nd	3 × 10^6^	No	No
09-ufmt	F	21	No	21	HBeAg	nd	No	No
20-ufmt	M	28	No	13	Anti-HBe	7 × 10^5^	No	No
26-ufmt	M	56	No	26	Anti-HBe	6 × 10^6^	Yes	No
27-ufmt	M	53	No	30	HBeAg	4 × 10^8^	No	No
29-ufmt	M	37	No	23	Anti-HBe	3 × 10^6^	No	No
								
*Genotype A2*								
02-hac	F	54	No	44	HBeAg	nd	Yes	L180M/M204V
32-ufmt	M	53	No	59	HBeAg	3 × 10^7^	Yes	L180M/M204V
42-ufmt	M	33	No	48	Anti-HBe	1 × 10^8^	Yes	L180M/M204V
41-ufmt	M	76	No	34	Anti-HBe	1 × 10^6^	No	No
								
*Genotype C*								
23-ufmt	F	25	No	51	HBeAg	2 × 10^7^	Yes	L180M/M204V
								
*Genotype D*								
39-ufmt	F	25	No	12	nd	4 × 10^7^	Yes	No
13-ufmt	M	68	No	40	HBeAg	nd	No	No
07-hac	M	57	No	36	Anti-HBe	1 × 10^8^	Yes	No
								
*Genotype F*								
01-ufmt	M	38	No	64	Anti-HBe	2 × 10^6^	Yes	L180M/M204I
								
*Genotype G*								
05-hgg	M	55	Yes	48	HBeAg/AntiHBe	5 × 10^6^	Yes	L180M/M204V
06-hac	M	46	No	51	Anti-HBe	3 × 10^6^	Yes	L180M/M204V
09-hgg	M	42	Yes	44	HBeAg	6 × 10^6^	Yes	L180M/M204V

nd, not determined

The genotype/subgenotype distribution of the HBV isolates showed a predominance of genotype A, subgenotype A1 (Table 1). Noteworthy, three isolates belonged to genotype G, a genotype which had never been described in Brazil. Genotypes A2, C, D and F were also identified.

The deduced amino acid sequences of the HBV DNA polymerase demonstrated that 20/32 (62%) isolates had lamivudine resistance mutations. The proportion of mutants varied from 60% to 100%, depending on the genotype, with the exception of genotype D, for which none of the three isolates was mutated. Fifteen patients showed the well-known double rtL180M/rtM204V mutation. Two others (03-hgg and 08-hgg) displayed a third mutation (rtV173L) related to lamivudine resistance [32]. At last, three patients displayed a single (rtM204I, patient 05-hac) or double (rtL180I/rtM204I, 11-hgg and 01-ufmt) mutation leading to the YIDD motif.

Patients infected with isolates showing lamivudine resistance mutations had HBV loads varying from 8 × 10^4 ^to 2 × 10^8 ^copies/mL (mean 2.9 × 10^7^, median 5 × 10^6^). Interestingly, values not very different (7 × 10^5 ^to 4 × 10^8 ^copies/mL [mean 6.9 × 10^7^, median 4.5 × 10^6^]) were found in treated patients without mutations. Median values of virus load were significantly higher in patients with HBeAg (2 × 10^7^copies/mL) than in patients with anti-HBe antibodies (1 × 10^6 ^copies/mL) (p < 0.05).

Failure to lamivudine treatment (phenotypic resistance) was evaluated by medical staff based in rapid increase of serum alanine aminotransferase (ALT) levels, sometimes accompanied by detection of a rebound of HBV load. There was a good correlation between phenotypic resistance and detection of lamivudine mutations. Indeed, 20/24 (83%) patients showing phenotypic resistance displayed lamivudine resistant mutations, whereas 8/8 (100%) who had no medical signs did not (Table 1).

### Complete sequencing and phylogenetic analysis of genotype G isolates

As mentioned above, 3/36 patients were infected with the rare HBV genotype G (HBV/G). All three were men who reported sexual relationships with men. Two of them were co-infected with HIV and were HBeAg positive: patient 09-hgg had very high levels of HBeAg, and was negative for anti-HBe, whereas patient 05-hgg had low levels of HBeAg and anti-HBe. The third patient (06-hac) was anti-HBe positive (Table 1).

**Figure 1:**
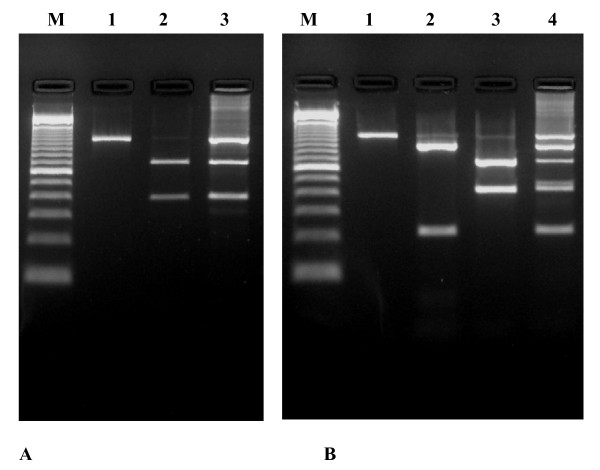
PCR-RFLP analysis of the pre-S/S genomic region. (A), *Eco*RI restriction endonuclease digestion. Lane 1, PCR products without *Eco*RI site (pattern predicted for genotype G). Lane 2, PCR products containing one *Eco*RI site (pattern deduced for most HBV genotype A isolates). Lane 3, PCR products derived from sample 09-hgg. (B), digestion with *Bam*HI. Lane 1, PCR products without *Bam*HI site (pattern predicted for genotype G). Lane 2, PCR products containing a *Bam*HI restriction site at nt 491 (pattern predicted for genotypes B and C and some genotype A isolates). Lane 3, PCR products containing a *Bam*HI site at nt 28 (subgenotype Aa and genotype G). Lane 4, PCR products derived from sample 09-hgg.

HBV/G has been commonly found in patients coinfected with an HBV isolate belonging to genotype A [12]. Whether the three HBV/G isolates from this study were associated with an HBV isolate belonging to another genotype was evaluated by PCR-RFLP analysis of the pre-S/S genomic region. Digestion of PCR products derived from sample 09-hgg with *Eco*RI restriction endonuclease (Fig. 1A, lane 3) showed the presence of two HBV populations, one without *Eco*RI site (pattern predicted for genotype G, shown in lane 1) and the other with one *Eco*RI site (pattern deduced for most HBV genotype A isolates, shown in lane 2). A similar experiment performed with *Bam*HI restriction enzyme (Fig. 1B) suggested a coinfection of patient 09-hgg with three HBV isolates, including G and A2. Furthermore, restriction digests of the two other HBV/G isolates from this study (05-hgg and 06-hac) showed the presence of additional, faint DNA bands, not explainable by incomplete digestion, but suggesting the occurrence of coinfection with low titers of HBV genotypes other than G. Such bands were not observed with isolates not belonging to genotype G.

**Figure 2:**
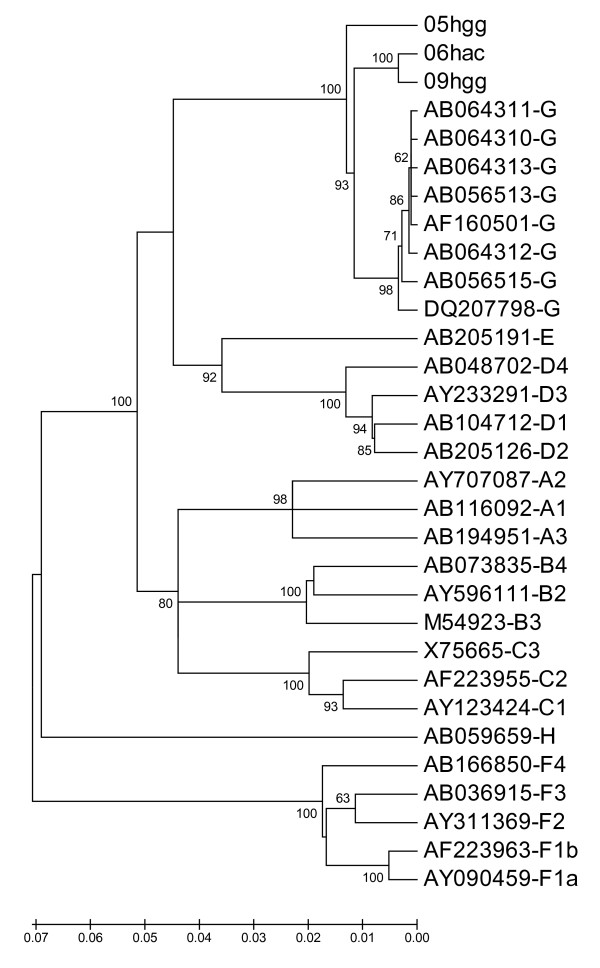
Phylogenetic tree based on full-length nucleotide sequences. Isolates from this work are named 05-hgg, 06-hac and 09-hgg. The other isolates are indicated by their GenBank accession number, followed by genotype/subgenotype designation. The horizontal bar shows a genetic distance scale.

Complete nucleotide sequences of the three HBV/G isolates (09-hgg, 05-hgg and 06-hac) were determined. All three had 3248 bp and showed a 36-nt insertion at the 5' end of the C gene, a 3-nt deletion in the pre-S1 region, and stop codons at positions 2 and 28 of the pre-core region, i.e. all features characteristics of genotype G [4]. Phylogenetic trees based on the complete genome (Fig. 2) and on entire pre-S/S, pre-core/core and X (not shown) regions confirmed the classification of isolates 09-hgg, 05-hgg and 06-hac into genotype G. It was observed that the three Brazilian G (HBV/G-Br) isolates were divergent between them and from those described before. HBV/G-Br isolates had a mean sequence divergence (1.9 ± 0.2%) higher than that observed between the eight previously described HBV/G isolates from other geographic regions (0.4 ± 0.1%). Such a divergence was even higher (2.3 ± 0.2%) when pre-S/S region alone was analyzed. Genotypes F and H were the genotypes (other than G) most distant from the HBV/G-Br isolates (Table 2).

**Table 2:** Nucleotide sequence divergence between the three HBV/G isolates from this work and isolates representative of all HBV genotypes and subgenotypes

	Genotypes (subgenotypes)							
	
Genomic regions	A (A1–A3)	B (B2–B4)	C (C1–C3)	D (D1–D4)	E (1 isolate)	F (F1–F4)	G (8 isolates)	H (1 isolate)
Entire genome	12.4 ± 0.6	13.7 ± 0.7	13.5 ± 0.6	12.4 ± 0.6	11.8 ± 0.5	15.5 ± 0.7	1.6 ± 0.2	16.5 ± 0.8
Pre-S/S	7.2 ± 0.8	8.9 ± 1.0	10.3 ± 1.0	9.3 ± 0.6	9.4 ± 0.9	13.2 ± 1.1	2.4 ± 0.4	12.9 ± 1.1

Sequence divergences are expressed in percents

Deduced amino acid sequences of pre-S1 region were well conserved among all G isolates. The three G isolates belonged to subtype *adw2 *(aminoacid residues K^122^, P^127 ^and K^160^). Pre-S2 and S regions of isolates 06-hac and 09-hgg displayed several aminoacid variations that are present in the sequence consensus of genotypes H and F, whereas isolate 05-hgg showed aminoacid variations related to genotype A (Table 3). Variations T49 (pre-S2) and M195 (S) were observed in all three HBV/G-Br. The last one resulted from the lamivudine resistance mutation at rtL180M position.

**Table 3:** Amino acid variability of Pre-S2 and S regions

**Pre-S2**																	**S**									
	
**Genotype/Isolate**	**07**	**14**	**18**	**30**	**31**	**32**	**33**	**35**	**37**	**41**	**42**	**46**	**48**	**49**	**51**	**54**	**08**	**18**	**20**	**44**	**45**	**49**	**51**	**63**	**68**	**195**
**G**	A	N	K/T*	G	I/T	V	N/S	V	T	H	I	F	R/K	I	D	P	F	G	F	G	V/A	P	L	I	T	I
**05-hgg**	-	D	R	-	T	L	-	-	N	-	-	S	-	T	-	T	-	-	-	-	S	L	Q	T	I	M
**06-hac**	Q	-	-	E	T	Q	-	-	-	L	T	-	K	T	G	-	L	-	-	E	-	-	-	-	-	M
**09-hgg**	Q	-	-	E	T	A	E	A	-	L	T	-	K	T	G	-	L	V	C	E	-	-	-	-	-	M
**F**	Q	D	R	-/E	T	Q	-	A/-	-	L	T	-	K	T	G	T/M	L	V	C	-	T/L	-	Q	T	T	I
**H**	Q	D	R	E	T	Q	-	A	-	L	T	-	K	T	-	M	L	G	-	V	P	-	Q	T	T	I
**D**	T	D	R	-	T	-	-	-	-	-/P	-/L	-	-	-	-	-/L	-	G	-	-	T	L	Q	T	T	I
**E**	T	D	R	-	T	-	-	-	-	L	-	-	-	-	-	-	-	G	-	-	A	L	Q	T	I	I
**B**	T	D	R	-	T	-	-/S	A/-	N	A/S	-	-/L	K	T	-	-	L	G	-	-/E	T	L	Q	T	T/I	I
**C**	T	D	R	-	T	-	-	-	-	P/L	-	F	-	T/-	-	T/-	-	G	-	-	A	L/-	Q	T	I	I
**A**	-/N	D	R/K	-	T	-/L	-	A/-	-/N	-	-	S	-	T/-	-	T/-	-	G	-	-	A/S	L	Q	T	I	I

-: same amino acid as genotype G consensus; *When two amino acids are separated by a slash, the first one represents the consensus of the genotype while the second one was only describewd in few isolates

## Discussion

Lamivudine resistance is due to aminoacid substitutions in the YMDD motif of the rt domain of the HBV polymerase. The resistance occurs by replacement of a methionine residue at position rt204 by either valine (rtM204V) or isoleucine (rtM204I), accompanied or not by a substitution at position rt180 [33] and more rarely by a substitution at position rt173 [32,34]. The occurrence of mutations rtL180M and rtV173L has been associated with prolonged lamivudine treatment [26,35]. Single mutants in the YMDD motif (rtM204V/I) replicate substantially more slowly *in vitro *than the wild type. Mutations at positions rt180 and rt173 may thus act as compensatory changes which partially restore the replication fitness of the virus. The triple mutation rtV173L/rtL180M/rtM204V/I results in concomitant amino acid substitutions E164D and I195M in the small S protein. It has been shown that this triple mutant has a reduced *in vitro *affinity to anti-HBs antibodies, similar to what occurs with the hepatitis B vaccine escape mutant G145R [32,35].

Here, most patients under prolonged lamivudine treatment displayed HBV DNA in serum. Lamivudine resistant mutations were observed in 67% of the cases, while 33% did not display recognized resistance mutations. In a previous study, performed with HIV infected patients with occult HBV infection (HBsAg negative), the group of patients without resistance mutations had a lower mean viral load than patients with resistance mutations [36]. However, in the present study, where all patients were HBsAg positive, no significant viral load differences were observed between those with and without lamivudine resistance mutations. In another study involving HBsAg positive, HIV infected patients, almost 50% who were HBV DNA positive did not display lamivudine resistance mutations. Furthermore, high HBV load was observed in at least one of these patients [34]. More detailed investigations of these patients, including adherence to treatment and eventual presence of quasispecies with resistant strains as minor populations, should be conducted. Understanding the circumstances and the molecular mechanisms leading to the circulation in relative high titers of HBV isolates with or without lamivudine resistance after prolonged utilization of the drug, may help to guide future therapies.

Recent studies have suggested that the rate of lamivudine resistance was higher in patients infected with HBV genotype A than in those with genotype D [37,38]. Here it was not possible to correlate lamivudine resistant mutations and genotype. Large surveys should be conducted to clarify the role of HBV genotype in the response of treatment.

The three HBV/G isolates described here displayed lamivudine resistance mutations. In previous studies, no particular association has been made between HBV/G and lamivudine resistance. HBV/G was discovered in San Francisco [4], where a large community of homosexual men lives. In a recent study conducted in Mexico, HBV/G was found exclusively in men who have sex with men [13]. Here, the three HBV/G isolates infected homosexual men. This supports the hypothesis of a major dissemination of HBV/G isolates in homosexual men.

HBV/G displays two stop codons in the precore region, abolishing HBeAg synthesis [4]. Despite this, a large proportion of patients infected with HBV/G are positive for HBeAg. The reason is that HBV/G is usually found in coinfection with HBV isolates from other genotypes, mainly genotype A [12]. Until recently, it was uncertain whether HBV/G could be found in monoinfections. Strong evidence of HBV/G monoinfection was obtained in 2006 by Chudy and colleagues [15]. HBV/G replication increased markedly when coinfected with HBV isolates from other genotypes [39]. The dynamics of co-infection of HBV/G with other genotypes would indicate that coinfection with genotype A is much more advantageous for enhanced replication than that occurring with other genotypes, including genotype C. Indeed, coinfection with HBV/A is frequent in individuals infected with HBV/G [12,40]. Here, the three HBV/G infected patients displayed different HBeAg/anti-HBe status. Two were HBeAg positive. One of them (09-hgg) was strongly positive while the other (05-hgg) has low titres of both HBeAg, and anti-HBe antibodies. The third sample was anti-HBe positive. Despite this variability, all three HBV/G samples seem to be cases of coinfection. As stated before [39], monoinfections should produce low serum HBV DNA titers, almost at the limit of detection. Here it was possible to amplify complete HBV/G genomes, that could hardly be done in the case of HBV/G monoinfection. HBV/G isolates of the present work displayed lamivudine mutations. To determine if there exists an association between Brazilian HBV/G and lamivudine mutations, large surveys should be conducted among men who have sex with men.

The three HBV/G isolates characterized here had mean sequence variations (1.9 ± 0.2%) higher than those (0.4 ± 0.1%) observed between HBV/G isolates from other geographic regions. Phylogenetic trees based on pre-S/S, pre-core/core and X regions confirmed the classification of isolates 09-hgg, 05-hgg and 06-hac into genotype G. In the case of isolate 05-hgg, the presence of amino acids characteristics of genotype A in pre-S2 and S regions does not allow excluding the occurrence of a recombination event. Nucleotide sequencing of individual clones will be necessary to test this hypothesis.

## Conclusion

A high proportion (88%) of long term lamivudine treated patients were HBV DNA positive. Most samples displayed recognized lamivudine resistant mutations with viral loads sufficiently high to be transmissible. Genotypes C and G, rarely or never detected before in South America, were identified. Disparities in routes of transmission (genotype G seems to be linked to homosexual behavior) and in pathogenic properties (genotype C is more aggressive than others) might explain the presence of these genotypes in the population studied. All three HBV/G isolates detected here displayed lamivudine mutations and showed large genetic variations between them. Large surveys conducted in the population of men who have sex with men should be useful to better characterize HBV genotype G.

## Methods

### Patients and serological studies

The study group consisted of 36 HBV chronically infected, ambulatory patients (29 male, seven female, mean age, 47 years) who were submitted to long-term (12 to 84 months, mean 39, median 35 months) lamivudine treatment at three public Brazilian hospitals (Gaffrée e Guinle hospital and Alceu Carneiro hospital, Rio de Janeiro; Infectious Diseases Ambulatory, Federal University, Cuiabá, Mato Grosso) between 2002 and 2004. All the patients were HBsAg positive and anti-HBc positive. The protocol used was approved by the Fiocruz Ethical Committee. Thirteen patients were anti-HIV-1 positive by conventional serological tests, and lamivudine was administered in these patients as part of antiretroviral treatment. The 36 patients belonged to different risk groups for HBV and HIV infections. Twenty (56%) were homosexual or bisexual men. Six belonged to the blood transfusion risk group and three were infected through vertical transmission. No risk group was identified for seven patients.

All HIV positive patients demonstrated clinical signs of lamivudine resistance, evaluated by medical staff. HBV monoinfected patients were selected randomly. Serum samples from all patients were initially tested for anti-HBc, HBsAg and anti-HBs in the hospitals. These samples were not available, and new blood samples were collected, retested for the presence of HBsAg and anti-HBc by enzyme-linked immunosorbent assays (Hepanostika Uni-form Organon Teknika B.V., Boxtel, Holland) and further used for genomic studies. Most HBsAg positive samples were submitted to HBeAg and anti-HBe antibodies detection. This was performed by using the commercially Axsym system (Abbott Laboratories, Abbott Park, IL).

### DNA extraction and conditions of PCR assays

DNA was extracted from serum samples using phenol/chloroform after treatment of 250 μL of serum with 0.5 mg/mL of proteinase K in the presence of 0.2 M NaCl and 0.25% SDS for 4 hours at 37°C as reported previously [17]. After precipitation with ethanol, the pellet was dried and resuspended in 30 μL of distilled water. Semi-nested and nested PCR assays were performed. PCR primers are shown in Table 4. The first round of amplification was performed with 1 μL of extracted DNA and one unit of Taq DNA polymerase (Invitrogen, San Diego, CA) in a final volume of 25 μL. After an initial DNA denaturation for 3 min at 94°C, amplification was for 35 cycles at 94°C for 40 sec, 55°C for 1 min and 72°C for 2 min 30 sec, followed by a final elongation for 7 min at 72°C. The second round was performed with 1 μL of first round PCR product in a final volume of 50 μL under the following parameters: 95°C for 30 sec, 52°C for 40 sec, 72°C for 2 min for 30 cycles, followed by a final elongation for 7 min at 72°C. Ten microliters of amplification products were loaded on agarose gels, electrophoresed, stained with bromide and visualized under UV light.

**Table 4:** Oligonucleotides used in this study

PCR round	Name	Sequence and position	Fragment size (bp)
*Nucleotide sequencing of complete genomes*			
1^st^	P1 (sense)	GAAAGCTTGAGCTCTTCTTTTTCACCTCTGCCTAATCA, 1821–841	2241
	S2 (antisense)	GGGTTTAAATGTATACCCAAAGA, 841–819	
2^nd^	PS1 (sense)	CCATATTCTTGGGAACAAGA, 2826–2845	1236
	S2 (antisense)	GGGTTTAAATGTATACCCAAAGA, 841–819	
2^nd^	C5 (sense)	AGACCACCAAATGCCCCTATC, 2299–2319	703
	PS3 (antisense)	TCCTTGTTGGGATTGAAGTCCCA, 3002–2980	
1^st^	PS1 (sense)	CCATATTCTTGGGAACAAGA, 2826–2845	1753
	X3 (antisense)	AGCAGCCATGGAAAGGAGGT, 1383–1363	
2^nd^	S18 (sense)	GGATGATGTGGTATTGGGGGCCA, 743–765	648
	X3 (antisense)	AGCAGCCATGGAAAGGAGGT, 1383–1363	
1^st^	S18 (sense)	GGATGATGTGGTATTGGGGGCCA, 743–765	1724
	C2 (antisense)	CTAACATTGAGATTCCCGAGATTGAGA, 2458–2432	
2^nd^	X1 (sense)	ACCTCCTTTCCATGGCTGCT, 1363–1383	712
	C3 (antisense)	TTGCCTGAGTGCAGTATGGT, 2056–2075	
2^nd^	PC1 (sense)	GGCTGTAGGCATAAATTGGTCTG, 1781–1803	667
	C2 (antisense)	CTAACATTGAGATTCCCGAGATTGAGA, 2458–2432	
			
*Other sequencing and PCR-RFLP experiments*			
1^st^	PS1	CCATATTCTTGGGAACAAGA, 2826–2845	1236
	S2	GGGTTTAAATGTATACCCAAAGA, 841–819	
	S22	GTATTTAAATGGATACCCACAGA, 841–819	
2^nd^	PS1	CCATATTCTTGGGAACAAGA, 2826–2845	1099
	SR	CGAACCACTGAACAAATGGC,704–685	
2^nd^	S4	TGCTGCTATGCCTCATCTTCT, 416–436	425
	S2	GGGTTTAAATGTATACCCAAAGA, 841–819	
	S22	GTATTTAAATGGATACCCACAGA, 841–819	

### Quantification of HBV DNA

HBV DNAs were quantified by real-time using the TaqMan technology, according to [41] with some modifications. A panel of reference sera was used for quantification. This panel, kindly supplied by Dr. Ikuta (Simbios biotecnologia, Lutherian University, Canoas, RS, Brazil), has been calibrated against commercially available panels (Optiquant HBV viral DNA, Acrometrix), and contained known numbers of HBV DNA molecules. Amplification assays were performed in a final volume of 25 μL of TaqMan universal MasterMix (Applied BioSystems, Foster City, CA) containing 2 μL of extracted DNA, 1 μM of each sense (5'-GGACCCCTGCTC GTGTTACA-3' nt 184–203) and antisense (5'-AGAGAAGTCCACCMCGAGTCTAGA-3', nt 273–249) primers, as well as 0.3 μM of probe (5'-FAM-TGTTGACAARAATCCT CACAATACCRCAGA-TAMRA-3', nt 218–247). After initial incubation steps of 2 min at 50°C and 10 min at 95°C, the PCR cycling program consisted of 50 cycles of 15 s at 95°C and 60 s at 60°C. Reactions were performed in a 7700 SDS system (Applied BioSystems). The assay has a limit of detection of 10 copies/reaction, i.e. 100 copies/mL of serum.

### Nucleotide Sequencing

PCR products of the partial S genomic region were directly sequenced. These products were obtained by semi-nested PCR amplification using primers PS1, S2 and S22 in the first round and PS4, S2 and S22 in the second round (Table 4). Complete nucleotide sequences of three genotype G isolates were determined after amplification by using primers shown in Table 4. Amplification products (50 μL) were electrophoresed on 2% agarose gels, DNA bands were extracted, and nucleotide sequences were determined using the BigDye Terminator kit (Applied Biosystems) and the same primers used for PCR amplification. Sequencing reactions were analyzed on an ABI373 automated sequencer (Applied Biosystems). The nucleotide sequences determined in this work have been deposited in the GenBank database under the accession numbers EF464070 to EF464099. Bioinformatics analysis of the sequences was performed applying the University of Wisconsin Genetic Computer Group package. Neighbor-joining phylogenetic trees were drawn and rearranged using the Mega program version 3 [42]. Representative sequences of each HBV genotype/subgenotype were selected after alignment of 884 full-length (>3100 nt) sequences [43].

### Genotyping by PCR-RFLP

A previously published PCR-RFLP genotyping method [6,19], based upon pre-S/S genomic region, was used to assess HBV coinfections with genotype G and other isolates. Required semi-nested PCR products were obtained with the use of primers PS1, S2 and S22 in the first round and PS1 and SR in the second round (Table 4).

## Authors' contributions

MB carried out the sequencing experiments. FJDS, KMRO, MA and CEB were involved in the clinical evaluation of the patients and supervised antiretroviral treatment. CN participated to the study design and revised the final version of the manuscript. SAG conceived and coordinated the study and wrote the manuscript. All authors read and approved the final version of the manuscript.

## References

[B1] Shepard CW, Simard EP, Finelli L, Fiore AE, Bell BP (2006). Hepatitis B virus infection: epidemiology and vaccination. Epidemiol Rev.

[B2] Arauz-Ruiz P, Norder H, Robertson BH, Magnius LO (2002). Genotype H: a new Amerindian genotype of hepatitis B virus revealed in Central America. J Gen Virol.

[B3] Norder H, Courouce AM, Magnius LO (1994). Complete genomes, phylogenetic relatedness, and structural proteins of six strains of the hepatitis B virus, four of which represent two new genotypes. Virology.

[B4] Stuyver L, De Gendt S, Van Geyt C, Zoulim F, Fried M, Schinazi RF, Rossau R (2000). A new genotype of hepatitis B virus: complete genome and phylogenetic relatedness. J Gen Virol.

[B5] Schaefer S (2007). Hepatitis B virus taxonomy and hepatitis B virus genotypes. World J Gastroenterol.

[B6] Araujo NM, Mello FC, Yoshida CF, Niel C, Gomes SA (2004). High proportion of subgroup A' (genotype A) among Brazilian isolates of Hepatitis B virus. Arch Virol.

[B7] Bowyer SM, van Staden L, Kew MC, Sim JG (1997). A unique segment of the hepatitis B virus group A genotype identified in isolates from South Africa. J Gen Virol.

[B8] Makuwa M, Souquiere S, Telfer P, Apetrei C, Vray M, Bedjabaga I, Mouinga-Ondeme A, Onanga R, Marx PA, Kazanji M, Roques P, Simon F (2006). Identification of hepatitis B virus subgenotype A3 in rural Gabon. J Med Virol.

[B9] Kurbanov F, Tanaka Y, Fujiwara K, Sugauchi F, Mbanya D, Zekeng L, Ndembi N, Ngansop C, Kaptue L, Miura T, Ido E, Hayami M, Ichimura H, Mizokami M (2005). A new subtype (subgenotype) Ac (A3) of hepatitis B virus and recombination between genotypes A and E in Cameroon. J Gen Virol.

[B10] Schaefer S (2005). Hepatitis B virus: significance of genotypes. J Viral Hepat.

[B11] Sanchez LV, Maldonado M, Bastidas-Ramirez BE, Norder H, Panduro A (2002). Genotypes and S-gene variability of Mexican hepatitis B virus strains. J Med Virol.

[B12] Kato H, Orito E, Gish RG, Sugauchi F, Suzuki S, Ueda R, Miyakawa Y, Mizokami M (2002). Characteristics of hepatitis B virus isolates of genotype G and their phylogenetic differences from the other six genotypes (A through F). J Virol.

[B13] Sanchez LV, Tanaka Y, Maldonado M, Mizokami M, Panduro A (2007). Difference of hepatitis B virus genotype distribution in two groups of mexican patients with different risk factors. High prevalence of genotype H and G. Intervirology.

[B14] Kato H, Orito E, Gish RG, Bzowej N, Newsom M, Sugauchi F, Suzuki S, Ueda R, Miyakawa Y, Mizokami M (2002). Hepatitis B e antigen in sera from individuals infected with hepatitis B virus of genotype G. Hepatology.

[B15] Chudy M, Schmidt M, Czudai V, Scheiblauer H, Nick S, Mosebach M, Hourfar MK, Seifried E, Roth WK, Grunelt E, Nübling CM (2006). Hepatitis B virus genotype G monoinfection and its transmission by blood components. Hepatology.

[B16] Kato H, Sugauchi F, Ozasa A, Kato T, Tanaka Y, Sakugawa H, Sata M, Hino K, Onji M, Okanoue T, Tanaka E, Kawata S, Suzuki K, Hige S, Ohno T, Orito E, Ueda R, Mizokami M (2004). Hepatitis B virus genotype G is an extremely rare genotype in Japan. Hepatol Res.

[B17] Niel C, Moraes MT, Gaspar AM, Yoshida CF, Gomes SA (1994). Genetic diversity of hepatitis B virus strains isolated in Rio de Janeiro, Brazil. J Med Virol.

[B18] Moraes MT, Gomes SA, Niel C (1996). Sequence analysis of pre-S/S gene of hepatitis B virus strains of genotypes A, D, and F isolated in Brazil. Arch Virol.

[B19] Motta-Castro AR, Martins RM, Yoshida CF, Teles SA, Paniago AM, Lima KM, Gomes SA (2005). Hepatitis B virus infection in isolated Afro-Brazilian communities. J Med Virol.

[B20] Benhamou Y, Katlama C, Lunel F, Coutellier A, Dohin E, Hamm N, Tubiana R, Herson S, Poynard T, Opolon P (1996). Effects of lamivudine on replication of hepatitis B virus in HIV-infected men. Ann Intern Med.

[B21] Liaw YF (2002). Management of YMDD mutations during lamivudine therapy in patients with chronic hepatitis B. J Gastroenterol Hepatol.

[B22] Gauthier J, Bourne EJ, Lutz MW, Crowther LM, Dienstag JL, Brown NA, Condreay LD (1999). Quantitation of hepatitis B viremia and emergence of YMDD variants in patients with chronic hepatitis B treated with lamivudine. J Infect Dis.

[B23] Lai CL (2004). Therapeutic advances in chronic hepatitis B. J Gastroenterol Hepatol.

[B24] Torresi J (2002). The virological and clinical significance of mutations in the overlapping envelope and polymerase genes of hepatitis B virus. J Clin Virol.

[B25] Jardi R, Buti M, Rodriguez-Frias F, Cotrina M, Costa X, Pascual C, Esteban R, Guardia J (1999). Rapid detection of lamivudine-resistant hepatitis B virus polymerase gene variants. J Virol Methods.

[B26] Yeh CT, Chien RN, Chu CM, Liaw YF (2000). Clearance of the original hepatitis B virus YMDD-motif mutants with emergence of distinct lamivudine-resistant mutants during prolonged lamivudine therapy. Hepatology.

[B27] Hoff J, Bani-Sadr F, Gassin M, Raffi F (2001). Evaluation of chronic hepatitis B virus (HBV) infection in coinfected patients receiving lamivudine as a component of anti-human immunodeficiency virus regimens. Clin Infect Dis.

[B28] Thibault V, Benhamou Y, Seguret C, Bochet M, Katlama C, Bricaire F, Opolon P, Poynard T, Agut H (1999). Hepatitis B virus (HBV) mutations associated with resistance to lamivudine in patients coinfected with HBV and human immunodeficiency virus. J Clin Microbiol.

[B29] Liaw YF, Chien RN, Yeh CT, Tsai SL, Chu CM (1999). Acute exacerbation and hepatitis B virus clearance after emergence of YMDD motif mutation during lamivudine therapy. Hepatology.

[B30] Durantel D, Brunelle MN, Gros E, Carrouee-Durantel S, Pichoud C, Villet S, Trepo C, Zoulim F (2005). Resistance of human hepatitis B virus to reverse transcriptase inhibitors: from genotypic to phenotypic testing. J Clin Virol.

[B31] Jacobson IM (2006). Therapeutic options for chronic hepatitis B: considerations and controversies. Am J Gastroenterol.

[B32] Torresi J, Earnest-Silveira L, Deliyannis G, Edgtton K, Zhuang H, Locarnini SA, Fyfe J, Sozzi T, Jackson DC (2002). Reduced antigenicity of the hepatitis B virus HBsAg protein arising as a consequence of sequence changes in the overlapping polymerase gene that are selected by lamivudine therapy. Virology.

[B33] Bartholomew MM, Jansen RW, Jeffers LJ, Reddy KR, Johnson LC, Bunzendahl H, Condreay LD, Tzakis AG, Schiff ER, Brown NA (1997). Hepatitis-B-virus resistance to lamivudine given for recurrent infection after orthotopic liver transplantation. Lancet.

[B34] Cooley L, Ayres A, Bartholomeusz A, Lewin S, Crowe S, Mijch A, Locarnini S, Sasadeusz J (2003). Prevalence and characterization of lamivudine-resistant hepatitis B virus mutations in HIV-HBV co-infected individuals. Aids.

[B35] Roque-Afonso AM, Ferey MP, Mackiewicz V, Fki L, Dussaix E (2003). Monitoring the emergence of hepatitis B virus polymerase gene variants during lamivudine therapy in human immunodeficiency virus coinfected patients: performance of CLIP sequencing and line probe assay. Antivir Ther.

[B36] Sucupira MV, Mello FC, Santos EA, Niel C, Rolla VC, Arabe J, Gomes SA (2006). Patterns of hepatitis B virus infection in Brazilian human immunodeficiency virus infected patients: high prevalence of occult infection and low frequency of lamivudine resistant mutations. Mem Inst Oswaldo Cruz.

[B37] Kobayashi M, Suzuki F, Akuta N, Suzuki Y, Arase Y, Ikeda K, Hosaka T, Sezaki H, Kobayashi M, Iwasaki S, Sato J, Watahiki S, Miyakawa Y, Kumada H (2006). Response to long-term lamivudine treatment in patients infected with hepatitis B virus genotypes A, B, and C. J Med Virol.

[B38] Orito E, Fujiwara K, Tanaka Y, Yuen MF, Lai CL, Kato T, Sugauchi F, Kusakabe A, Sata M, Okanoue T, Niitsuma H, Sakugawa H, Hasegawa I, Mizokami M (2006). A case-control study of response to lamivudine therapy for 2 years in Japanese and Chinese patients chronically infected with hepatitis B virus of genotypes Bj, Ba and C. Hepatol Res.

[B39] Sugiyama M, Tanaka Y, Sakamoto T, Maruyama I, Shimada T, Takahashi S, Shirai T, Kato H, Nagao M, Miyakawa Y, Mizokami M (2007). Early dynamics of hepatitis B virus in chimeric mice carrying human hepatocytes monoinfected or coinfected with genotype G. Hepatology.

[B40] Osiowy C, Giles E (2003). Evaluation of the INNO-LiPA HBV genotyping assay for determination of hepatitis B virus genotype. J Clin Microbiol.

[B41] Pas SD, Niesters HG (2002). Detection of HBV DNA using real time analysis. J Clin Virol.

[B42] Kumar S, Tamura K, Nei M (2004). MEGA3: Integrated software for Molecular Evolutionary Genetics Analysis and sequence alignment. Brief Bioinform.

[B43] Kay A, Zoulim F (2007). Hepatitis B virus genetic variability and evolution. Virus Res.

